# The complete chloroplast genome of *Dendrobium comatum* (Orchidaceae)

**DOI:** 10.1080/23802359.2021.1990152

**Published:** 2021-10-15

**Authors:** Feng Liu, Xu Xiao, Mingtai An, Zhi Li

**Affiliations:** College of Forestry, Guizhou University, Guiyang, China

**Keywords:** *Dendrobium comatum*, chloroplast genome, high-throughput sequencing, phylogenomic analysis

## Abstract

*Dendrobium comatum* is a rare orchid plant. To further its research and conservation, we reported and characterized its complete chloroplast (cp) genome by using Illumina paired-end sequencing data. We also analyzed its characteristics and studied its clustering relationships. The total chloroplast genome size was 158,008 bp, including inverted repeats (IRs) of 27,032 bp separated by a large single copy (LSC) region and a small single copy (SSC) region of 85,592 and 18,352 bp, respectively. A total of 132 genes, including 38 tRNA, 8 rRNA, and 87 protein-coding genes, were identified. Phylogenetic analysis showed that *D. comatum* is sister to *D. kingianum* with sufficient support.

The genus *Dendrobium* is one of the three largest genera in the family Orchidaceae (Xiang et al. 2013), comprising approximately 1100 species that are mainly distributed from India to Japan; Malaysia and Indonesia to the south; and Australia, New Guinea, and the Pacific islands to the east. There are 78 species (14 endemic) in 14 sections in China, some of which have been extensively used as traditional Chinese medicine (TCM) (Chen et al. 2009; Tang et al. 2020). However, many wild *Dendrobium* species are in extreme danger of extinction due to overharvesting and habitat destruction. *Dendrobium comatum* (Blume) Lindl (1830) belongs to the genus *Dendrobium* (Orchidaceae: Epidendroideae) and is distributed in South and East China. This species is characterized by a terminal pseudobulb comprising 2 or 3 internodes; the mid-lobe of the lip is very plicate, deeply fimbriate-lacerate, and divided into many fine segments (Chen et al. 2009). Its distribution range is narrow, and it has extremely high medicinal value. *D. comatum* is listed as an endangered species on the China Species Red List and as EN on the IUCN (Wang and Xie 2004). In this paper, the complete chloroplast genome of *D. comatum* was constructed from whole-genome sequencing data to provide molecular data for the systematic investigation of Illumina Orchidaceae.

Five plants transplanted from a wild area in Pingtang, which is located in Pingtang County, Guizhou Province, China (25°45′39″N, 107°17′59″E, Alt. 823 m), were collected for morphological observations and other studies. For the DNA extraction of *Dendrobium comatum*, 2–3 g of fresh leaves were harvested. The specimen and extracted DNA were deposited at Biodiversity Research Laboratory, Forestry College of Guizhou University (http://fc.gzu.edu.cn) under the voucher number LZ20200501 (collected by Zhi Li, lizhighz@163.com). Total genomic DNA was extracted from fresh leaves by using the modified CTAB procedure of Doyle and Doyle (1987) and sequenced on the Illumina NovaSeq6000 platform (Illumina, San Diego, CA). Genome sequences were identified and assembled with SPAdes v.3.5.0 (Lapidus et al. 2014).

The cp genome sequence of *D. comatum* (GenBank accession MZ666386**)** is 158,008 bp in size and contains a pair of inverted repeats (IRs) of 27,032 bp, which are separated by a large single copy (LSC) region of 85,592 bp and a small single copy (SSC) region of 18,352 bp. The cp genome contains 132 genes, including 86 protein-coding genes, 38 transfer RNA genes, and eight ribosomal RNA genes. The overall GC content of the cp genome is 37.12%.

To perform a phylogenomic analysis, twenty-seven complete chloroplast genomes within the *Dendrobium* genus were considered along with *D. comatum*. Two species from the *Goodyera* genus (*Goodyera velutina* and *Goodyera procera*) were selected as outgroups because *Goodyera* belongs to a clade that is sister to *Dendrobium.* The chloroplast genome sequences were aligned using MAFFT (Katoh and Standley 2013). Phylogenetic analysis using the maximum likelihood algorithm was conducted with RAxML8.1.5 (Kalyaanamoorthy et al. 2017) implemented in Geneious ver. 10.1 (http://www.geneious.com, Kearse et al. 2012), and a maximum-likelihood (ML) phylogeny was used to construct a phylogenetic relationship tree. The results showed that *D. comatum* is phylogenetically related to *Dendrobium kingianum.* This newly reported cp genome of *Dendrobium comatum* will be helpful for further investigation of the taxonomy and phylogeny of *Dendrobium* (Figure 1).

**Figure 1.**
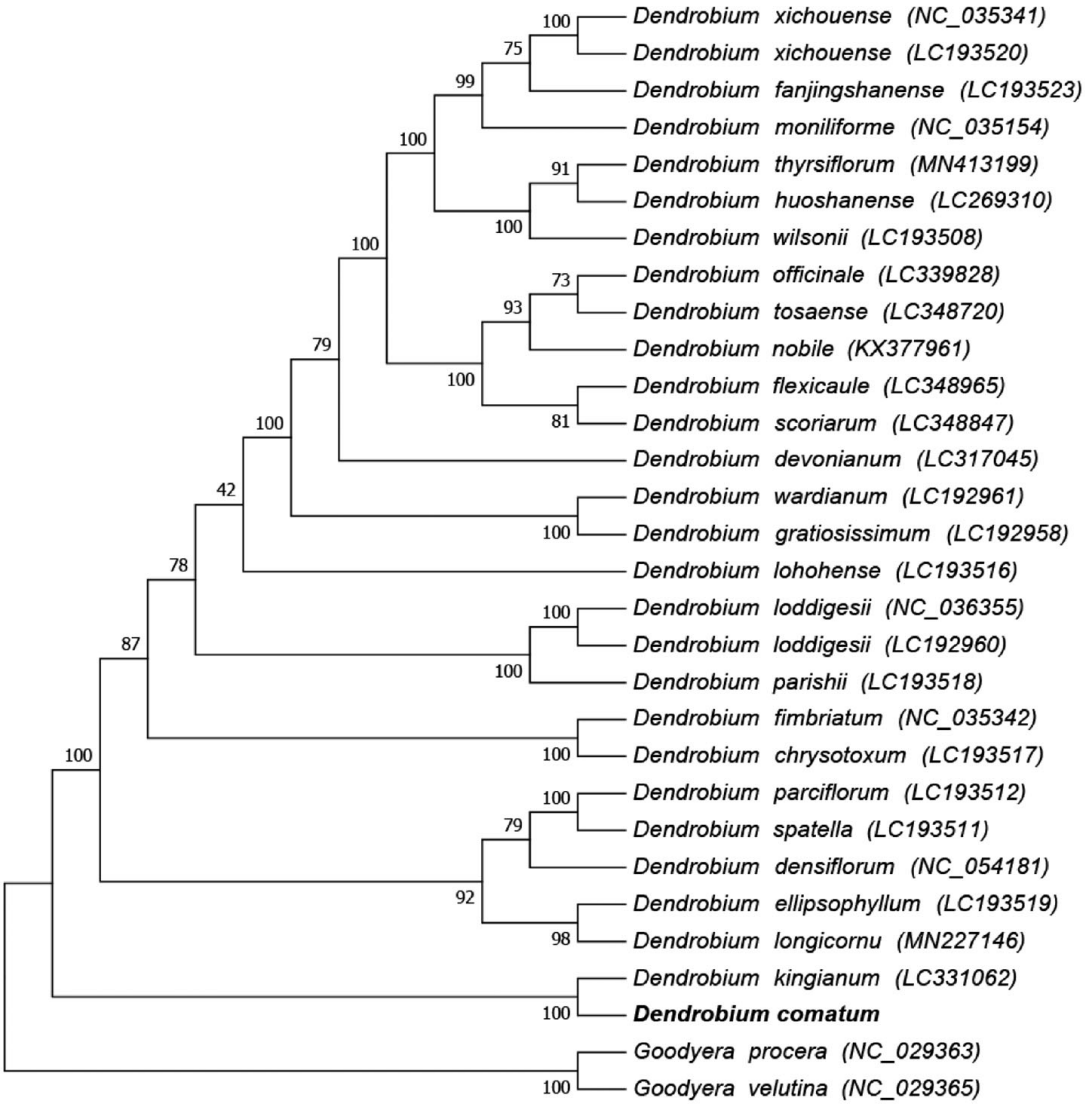
Phylogenetic relationships of 29 species based on the maximum-likelihood (ML) analysis of chloroplast genomes.

## Data Availability

The data used to support the findings of this study are available from the corresponding author upon request and in GenBank at [https://www.ncbi.nlm.nih.gov/genbank/] (accession number MZ666386). The associated BioProject, SRA, and Bio-Sample numbers are PRJNA759294, SRR15694584, and SAMN21163048 respectively.

## References

[CIT0001] Chen SC, Liu ZJ, Zhu GH, Lang KY, Ji ZH, Luo YB, Jin XH, Cribb PJ, Wood JJ, Gale SW, et al. 2009. Orchidaceae. In: Wu ZY, Raven PH, Hong DY, editors. Flora of China. Vol. 25. St. Louis (MO): Missouri Botanical Garden Press; p. 1–506.

[CIT0002] Doyle JJ, Doyle JL. 1987. A rapid DNA isolation procedure from small quantities of fresh leaf tissue. Phytochem Bull. 19:11–15.

[CIT0003] Kalyaanamoorthy S, Minh BQ, Wong TKF, von Haeseler A, Jermiin LS. 2017. ModelFinder: fast model selection for accurate phylogenetic estimates. Nat Methods. 14(6):587–589. 28481363 10.1038/nmeth.4285 PMC5453245

[CIT0004] Katoh K, Standley DM. 2013. MAFFT multiple sequence alignment software version 7: improvements in performance and usability. Mol Biol Evol. 30(4):772–780. 23329690 10.1093/molbev/mst010 PMC3603318

[CIT0005] Kearse M, Moir R, Wilson A, Stones-Havas S, Cheung M, Sturrock S, Buxton S, Cooper A, Markowitz S, Duran C, et al. 2012. Geneious Basic: an integrated and extendable desktop software platform for the organization and analysis of sequence data. Bioinformatics. 28(12):1647–1649. 22543367 10.1093/bioinformatics/bts199 PMC3371832

[CIT0006] Lapidus A, Antipov D, Bankevich A, Gurevich A, Korobeynikov A, Nurk S, Prjibelski A, Safonova Y, Vasilinetc I, Pevzner PA. 2014. New frontiers of genome assembly with SPAdes 3.0. (poster).

[CIT0007] Tang Y, Yang J, Niu Z, Ding X. 2020. The complete chloroplast genome sequence of a traditional chinese medicine plant *bulbophyllum disciflorum* rolfe (orchidaceae). Mitochondrial DNA B. 5 (1):59–60. 10.1080/23802359.2019.1670112 PMC7720722 33366421

[CIT0008] Wang S, Xie Y. 2004. China species red list. RedList. Vol. 1. Beijing (China): Higher Education Press.

[CIT0009] Xiang X-G, Schuiteman A, Li D-Z, Huang W-C, Chung S-W, Li J-W, Zhou H-L, Jin W-T, Lai Y-J, Li Z-Y, et al. 2013. Molecular systematics of *Dendrobium* (Orchidaceae, Dendrobieae) from mainland Asia based on plastidand nuclear sequences. Mol Phylogenet Evol. 69(3):950–960. 23811435 10.1016/j.ympev.2013.06.009

